# The Causation of Epidemic Diarrhœa

**Published:** 1906-02-24

**Authors:** 


### HOSPITAL CLINICS.

### THE CAUSATION OF EPIDEMIC DIARRHŒA.

An important contribution to the study of this subject is contained in an article by Dr. J. E. Sandilands in the January issue of the "Journal of Hygiene." This author acknowledges the immense amount of work on the bacterial content and the chemical composition of cow's milk which has been undertaken with a view to clearing up the intimate pathology of epidemic diarrhœa; but, as will be seen, he is disposed to traverse the generally accepted view that bacterial decomposition *per se* is an efficient cause of epidemic diarrhœa in infants. Park, for instance, has demonstrated that the bacterial content of milk varies with the temperature, and that a sample which held bacteria to the number of 30,000 per cubic centimetre, contained, after standing for 24 hours at a temperature of 35° C., no fewer than 25,000,000,000. But, asks Dr. Sandilands, are these enormous numbers of putrefactive bacteria really harmful? The balance of opinion among authorities has undoubtedly been in favour of the view that bacterial decomposition of milk is quite sufficient to convert the latter into an infant poison. Park states that "children in cities sicken on the milk supplied in summer, that those put on milk which is sterile or contains only a few bacteria as a rule mend rapidly, while those kept on impure milk continue ill or die." Dr. Newman, again, holds "that stale milk contains toxic properties altogether apart from, and in addition to, bacteria. It is possible that the products of organismal action have a much greater effect in the causation of diarrhœa than is generally supposed." Delépine states that "long keeping and high temperature are the two important factors which determine whether a sample of infected milk will contain a sufficient quantity of bacteria or bacterial products to produce infection." It is clear, therefore, that most people believe in the relation between a high bacterial content of the milk sold in cities and the occurrence of epidemic diarrhœa.

In the course of his investigation Dr. Sandilands was struck with the fact that infants fed upon condensed milk are three times more liable to fatal diarrhœa than they should be on the assumption of an average distribution of the disease in a severe form regardless of diet. In the case of children fed upon cow's milk, the occurrence of fatal diarrhœa amounted to two and a half times the average. Now, in view of the fact that Nestlé's milk was the brand in use in 90 per cent. of the fatal cases of diarrhœa among infants in Finsbury and Brighton who were fed upon condensed milk, it seemed a matter of importance to make some experimental estimates of the bacterial content of this milk under varying conditions. For this purpose the author opened and exposed tins of the milk to the air of his laboratory for 24 hours. The temperature of the laboratory during the experiment ranged from 68° to 45° F. The exposed samples were then mixed and incubated, part at a temperature of 22° C. and part at body-heat.

The result showed that three contaminated samples gave no evidence of decomposition after incubation for 12 days at a temperature of 22° C., while two others incubated at body-heat behaved in a similar manner, none of these yielding a bacterial content of more than 3,000 per cubic centimetre in six days. But a specimen diluted ten times with distilled and sterile water gave a count of eleven millions of bacteria per cubic centimetre on the second day of incubation at body-heat. In this case there was marked rancidity. It therefore appears that, by comparison with cow's milk, the bacterial content of Nestlé's milk is low, and remains low for a week at a summer temperature of 70° F. Moreover, bacteria do not always multiply in this milk even at body-heat, and when they do multiply at a great pace they produce obvious evidence of unfitness for food in the shape of a rancid smell. Therefore, as the author observes, it is almost certain that even in hot summer weather a tin of condensed milk will be consumed before decomposition sets in, while, if decomposition should occur, the milk will become so altered as to be repugnant to the child, even though the change be overlooked by the mother. It appears certain, therefore, that a high bacterial content plays no part in the causation of the epidemic diarrhœa which is so fatal to children fed upon Nestlé's milk.

Turning next to the relation of the bacterial content of cow's milk to the occurrence of this epidemic diarrhœa, the author notes that the pathogenicity of cow's milk, as measured by its capacity to produce lesions, is not directly influenced by the temperature of the atmosphere. Tables are given to illustrate Ballard's law that the prevalence of diarrhœa is correlated with the earth temperature, and cannot therefore vary with that of the atmosphere. Thus, in 1858 a hot period at the beginning of summer was accompanied by less diarrhœa than a colder period at the end of the same summer, while a cold spell during June and July of 1865 resulted in three times the amount of diarrhœa which accompanied a warmer period in August and September of the same year.

The conclusion, therefore, remains that the incidence of diarrhœa is not directly influenced by the bacterial content of cow's milk or of Nestlé's milk, for if it were we should find a constant relationship between air-temperatures favourable to bacterial multiplication, and epidemic diarrhœa. In point of fact diarrhœa follows hot spells, and does not accompany them unless they be of long duration.

If, then, the bacterial content of Nestlé's milk cannot be invoked to explain away the fatalities from diarrhœa which accompany its use, it remains to search further for the cause. Dr. Sandilands has satisfied himself that the milk used in the preparation of Nestlé's product compares favourably in its quality and the care used in its collection, with the milk of English cows. Neither does he consider that the sugar by which it is preserved can be blamed for the untoward results which have been seen to follow its use. It is left, therefore, to consider the possible explanation afforded by the assumption that the milk is infected by virulent organisms after it has been opened—that is to say, infected in the homes of the users.

It is generally known that epidemic diarrhœa is highly infectious, and it has several times been observed that the distribution of an epidemic of this disease is focussed upon localities instead of following the distribution areas of various sources of supply. The significance of this observation is not obscure. It points to the local spread of a contagion as opposed to the dissemination of a food supply infected at its source. It means that epidemic diarrhœa is an infective disease originated by the dissemination of infected dejecta, an infection comparable both in its methods of access to the body and its lesions with that of enteric fever, and a disease to be attacked along the lines with which enteric fever has made us familiar—namely, by the disinfection of the fæces of victims of the disorder. As regards the actual vehicles of the infection, the author believes that the air is partly responsible, and that virulent organisms from dried fæces are probably blown about in great quantity. But another and more preventible source is suggested by a report upon the health of Brighton rendered by Dr. Newsholme. He says: "The sugar used in sweetening milk is often black with flies which have come from a neighbouring dust-bin or manure heap, or from the liquid stools of a diarrhœal patient in a neighbouring house. Flies have to be picked out of the half-empty can of condensed milk before the remaining contents can be used for the next meal. When we remember the personal uncleanliness of some mothers, and that they often prepare their infants' food with unwashed hands, the inoculation of this food with virulent colon bacilli ceases to be a matter of surprise."

Dr. Sandilands considers that the greater capacity for causing epidemic diarrhœa which is evidenced by Nestlé's milk as opposed to cow's milk is a result of the greater attraction to flies offered by the sugar in the former. It has long been known from the work of Nuttall that flies are competent carriers of the infection of cholera and of enteric fever, and the natural history of flies shows that they, like the disease in question, follow hot spells, and do not accompany them unless these latter be protracted.

The general conclusions of the author's investigations are as follows:—

1. Nestlé's milk, with a relatively small bacterial content, is a more frequent cause of epidemic diarrhœa than is cow's milk with a large one.

2. In certain seasons cow's milk may be highly bacterial, yet harmless.

3. The number of bacteria, whether in preserved or natural milk, has no direct bearing upon the production of epidemic diarrhœa.

4. The majority of instances of diarrhœa depend upon a local infection of the milk used—that is to say, an infection in the homes of its users.

5. This infection is fæcal.

6. The life history of flies, and their habits, make it likely that they are the vehicles of the contagion.

